# Development of the Signposting Questionnaire for Autism (SQ-A): measurement comparison with the 10-item Autism Spectrum Quotient-Child and the Strengths and Difficulties Questionnaire in the UK and Latvia

**DOI:** 10.1186/s13229-020-00368-9

**Published:** 2020-08-15

**Authors:** Catherine R. G. Jones, Sarah L. Barrett, Ieva Bite, Maria Legzdina, Kristina Arina, Andrea Higgins, Kyla Honey, Sarah J. Carrington, Dale Hay, Johanna Condon, Susan R. Leekam

**Affiliations:** 1 School of Psychology, Cardiff University, Cardiff, UK; 2 Department of Psychology, University of Latvia, Riga, Latvia; 3 Department of Psychology, School of Life and Health Sciences, Aston University, Birmingham, UK; 4 Healios Neurodevelopment Service, Healios Ltd, Southampton, UK

**Keywords:** Autism, Signposting Questionnaire for Autism, Autism Spectrum Quotient, Strengths and Difficulties Questionnaire, Diagnostic Interview for Social Communication Disorders, Signposting, Diagnosis, Parent report

## Abstract

**Background:**

Recognising the signs of autism spectrum disorder (ASD) can be a challenge for frontline professionals. The use of brief parent-completed questionnaires for recording the signs of ASD in school-aged children may be an important and efficient contributor to professional insight. However, to date, such questionnaires have not been designed to be used in coordination with current standardised Diagnostic and Statistical Manual of Mental Disorders (DSM-5) diagnostic tools. Furthermore, the measurement characteristics of such questionnaires have been unexplored across countries that differ in levels of national autism service provision and cultural interpretation of the signs of ASD.

**Methods:**

A new 14-item questionnaire (Signposting Questionnaire for Autism (SQ-A)) was developed using published DSM-5 items from a clinical interview, the Diagnostic Interview for Social Communication Disorders (DISCO). Measurement comparison was tested with the Short Autism Spectrum Quotient-Child (AQ-10) and the Strengths and Difficulties Questionnaire (SDQ). Parents of 4–11-year-old children in the UK (*N* = 200) and Latvia (*N* = 104) completed all three questionnaires. Information on clinical diagnosis provided by parents led to classification into three groups: ASD diagnosis, other conditions and no conditions. In the UK, a subsample of teachers also provided cross-informant reliability.

**Results:**

In both countries, there was evidence of acceptable to good internal consistency for the SQ-A, with significantly higher scores for the ASD group and evidence of convergent and discriminant validity. There was also good parent-teacher reliability for the three measures. Notably, the questionnaires designed specifically to measure autism (SQ-A, AQ-10) performed more similarly to one another compared to the broader SDQ, with differences found for the ASD group. The overall pattern of responding to the three questionnaires was highly similar between countries.

**Conclusions:**

These results indicate the potential of the 14-item SQ-A to guide frontline professionals in the recognition of the signs of autism in children, facilitating the provision of appropriate support.

## Background

Families can face long delays for a formal diagnosis of autism spectrum disorder (ASD)^1^ [1–4], and even in countries with well-established diagnostic services, most children are not diagnosed until their school years [5–7]. Countries without well-established diagnostic services and limited public and professional awareness may be further restricted in their capacity to provide timely support. Against this background, increasing awareness of the signs of autism among frontline professionals is imperative, so that they are better equipped to understand behaviours, regardless of whether a diagnostic assessment has occurred. Related to this, family engagement with education and health systems prior to diagnosis have been associated with a lower age of eventual diagnosis [8]. Brief questionnaires that identify the signs of ASD in school-aged children provide an efficient and accessible tool for increasing parent and professional recognition and understanding of the profile of a child’s behaviours. However, to date, brief questionnaires have not been designed according to the latest diagnostic criteria, using items specifically derived from the Diagnostic and Statistical Manual of Mental Disorders, fifth edition (DSM-5) [9] diagnostic tools. Furthermore, few have been shown to be valid across different cultures where the interpretation of autistic features might vary [10].

To address these issues, our study had three goals. The first was to develop a parent-report signposting questionnaire (Signposting Questionnaire for Autism (SQ-A)) that directly coordinates with a DSM-5-compatible standardised diagnostic interview measure and to test it against an existing autism measure (short version of Autism Spectrum Quotient-Children’s version; AQ-10 [11]) and a measure of general child psychopathology (Strengths and Difficulties Questionnaire (SDQ) [12]). The second was to address the issue of possible cultural differences by comparing the three questionnaires in the UK, primarily Wales, with Latvia, a country with very different provision for autism. The third was to contribute new evidence to research on the AQ-10 and the SDQ in different countries.

To achieve our first goal of developing and testing the SQ-A, we adapted a set of 14 highly discriminating items [13, 14] from an established clinical 320-item interview, the Diagnostic Interview for Social and Communication Disorders (DISCO) [15, 16], which has an 85-item DSM-5 algorithm [17] and an abbreviated 54-item DSM-5 algorithm [13]. As a first step in the evaluation of the SQ-A, we investigated how the questionnaire performed for children with ASD compared with children with other special educational needs but without ASD and those with no known difficulties. We expected the ASD group to score significantly more highly on the SQ-A than the other two non-ASD groups. A number of questionnaires already exist to support the identification of ASD in children, with many conceptualised as a “screening” tool. These include the Autism Spectrum Quotient-Children’s version (AQ-Child) [18] and its shorter 10-item version (AQ-10) [11], the Autism Spectrum Screening Questionnaire (ASSQ) [19], the Social Communication Disorders Checklist (SCDC) [20], and Social Communication Questionnaire (SCQ) [21] (see [22] for a review). The SQ-A is distinct in having the dual advantage of being derived from both a clinical diagnostic interview and being a short, reduced-item set that focusses on the most discriminating items. For example, whereas the SCQ is derived from the Autism Diagnostic Interview-Revised (ADI-R) [23] it is long at 40 items, and while the AQ-10 is appealingly brief at 10 items it was not originally derived from a specific diagnostic instrument [24]. Particularly, the SQ-A is unique in not only using identified DSM-5 items, but including only those DSM-5 items that are known to be highly discriminating [13, 14]. Lastly, the SQ-A has an advantage over other measures as it is derived from a nested set of measures, with potential benefits for both research and future clinical applications.

To assist in the development of the questionnaire and testing of its measurement, comparison was made with two other questionnaires. These comparisons provided information about both convergent and discriminant validity [25]. First, to meet the criteria of convergent validity, the SQ-A should correlate positively with another screening tool; thus, a comparison was made with the AQ-10 [11], a measure of similar length designed as a screening tool to specifically identify autistic features in children aged 4–11 years. Second, to establish discriminant validity between measures of autism compared to broader dimensions of childhood psychopathology, we compared the SQ-A with the SDQ [12], a widely used and well-validated measure of general psychopathology. There is evidence that ASD diagnosis is associated with high levels of behavioural and emotional difficulties assessed using the SDQ [26]. Furthermore, it is known that SDQ scores are elevated when ASD co-occurs with other neurodevelopmental conditions (e.g. [27]). This analysis permitted the identification of ASD-only and ASD-co-occurring subgroups within the ASD group.

Our second goal focussed on a comparison of the questionnaire data collected in the UK with data collected in Latvia. Autism was not recognised in Latvia as a diagnosis until the early 1990s [28], and clinical diagnosis is still conducted by only a few centres with access to a limited number of diagnostic measures. In contrast, the DSM model of autism diagnosis has been established in the UK since its earliest conception in 1980 [29]. Today, the four countries of the UK separately recognise and support autism at the level of government. Wales is guided by a decade-long national government strategy with systematic awareness-raising information campaigns and diagnostic services available at a population-wide level. Therefore, the current study enabled performance of the questionnaires to be compared in two countries with different levels of provision for autism services and likely differences in the interpretation, recognition and understanding of the signs of ASD.

Our final goal was to contribute new psychometric information about the AQ-10 and the SDQ in different countries. The AQ-10 has been validated [11], and although all age versions show good specificity and sensitivity, most studies using the AQ-10 have focussed only on the adult version. Furthermore, studies with children [11] have used only comparison samples from the typical population, which can potentially bias the evaluation of the assessment’s discriminatory power (see [30, 31]). This will be the first study of the AQ-10 Child version to specifically include parents of children with SEN as a comparison group. It will also be the first to include teacher data and the first study conducted in Latvia. To our knowledge, the study also contributes the first research findings on the SDQ in Latvia and is the first to provide a comparison between the SDQ and AQ-10 in both countries.

In summary, the study developed the SQ-A from the most highly discriminating items from the DISCO DSM-5 algorithm [14]. We report on its measurement comparison with other well-established questionnaires (AQ-10; SDQ) in two countries, the UK and Latvia, a country with limited support services for autism. We also provide new findings from both countries on the AQ-10 and SDQ.

## Study 1—children from the UK

### Method

#### Participants

Participants were parents or guardians of 4- to 11-year-old children primarily living in Wales, UK. Recruitment was through three convenience sampling methods. First, we contacted 10 primary schools in the South Wales area. For eight schools, we targeted parents of children on the SEN register, and for two schools, we targeted all parents. Second, we recruited parents of children with and without SEN via social media and snowballing, targeting parents/guardians aged 25–50 years in the South Wales area who had shown an interest in neurodevelopmental disorders. Finally, we also contacted the parents of autistic children through existing mailing lists (a university research participant database and a database belonging to the Welsh Local Government Association).

Using these three methods, data were collected from parents and guardians of 208 children. One child had > 10% missing data for more than one questionnaire, and seven were under the age of 4 years. These participants were removed from the main analysis, producing a final sample of *N* = 200. Children were categorised into three groups based on information provided by parents: *ASD* (clinical diagnosis of ASD; *N* = 102); *Other* (no diagnosis of ASD but on SEN register or had another neurodevelopmental, psychological or physical condition; *N* = 52) and *None* (not on SEN register and no known condition; *N* = 46).

To explore cross-informant reliability, we also collected data from teachers from a subset of the participants that were recruited through schools. A member of educational staff who knew the child well was invited to complete the questionnaires if a parent gave consent. Teacher data were collected for 39 of the 44 children recruited from schools. Four of these 39 children were under 4 years but were retained in the specific parent-teacher analyses to maximise sample size. Note that they were not included in any other analyses, which focussed on children aged 4–11 years. One teacher was missing > 10% data on more than one questionnaire so was excluded from the dataset (*N* = 38).

#### Materials

##### Demographic questions

Parents and teachers were both asked the following demographic questions about the child: date of birth, gender, SEN status. Parents were also asked if their child had a clinical diagnosis of ASD and/or any of the following: speech and language impairment, attention-deficit hyperactivity disorder, developmental coordination disorder, conduct disorder or oppositional defiant disorder, anxiety disorder, Tourette’s syndrome, a specific genetic disorder (e.g. Fragile X syndrome, Williams syndrome), intellectual (learning) disability, a physical disability, and hearing or visual impairment. Questions were asked about their child’s level of language; both expressive and receptive (see Table 1). In addition, parents were asked the following questions about themselves: relationship to the child, employment status, age at leaving education, nationality, location and native language. Some demographic questions were added during the data collection period (nationality, location, native language), resulting in some missing data.

**Table 1:** Demographic data for the whole UK sample and split by *ASD*, *Other* and *None* groups

	Whole sample(*N* = 200)	ASD (*N* = 102)	Other (*N* = 52)	None(*N* = 46)
Age (years)	7.97 (2.0)§	8.29 (1.96)‡	7.68 (2.04)†	7.57 (1.94)†
Gender	149 (74.5%) M† 50 (25%) F	86 (84.3%) M 16 (15.7%) F	38 (73.1%) M† 13 (25%) F	25 (54.3%) M 21 (45.7%) F
Language expression				
None	12 (6%)	9 (8.8%)	2 (3.8%)	0 (0%)
Single words	8 (4%)	6 (5.9%)	2 (3.8%)	0 (0%)
Simple phrases	31 (15.5%)	24 (23.5%)	7 (13.5%)	0 (0%)
Full sentences	149 (74.5%)	63 (61.8%)	41 (78.8%)	46 (100%)
Language comprehension				
None	0 (0%)	0 (0%)	0 (0%)	0 (0%)
Single words	7 (3.5%)	7 (6.9%)	0 (0%)	0 (0%)
Simple phrases	41 (20.5%)	30 (29.4%)	11 (21.2%)	0 (0%)
Full sentences	152 (76%)	65 (63.7%)	41 (78.8%)	46 (100%)

Standard deviation (SD) reported in brackets for age; percentage reported in brackets for gender, language use and language comprehension

†Missing = 1 participant

‡Missing = 2 participants

§Missing = 4 participants

##### SQ-A

The SQ-A was developed from the DISCO, a 320-item semi-structured clinical interview, with good inter-rater reliability and criterion validity [15, 32, 33] and good agreement with both the Autism Diagnostic Interview-Revised (ADI-R [23]) and Autism Diagnostic Observation Schedule (ADOS [34]) [32, 33]. An 85-item DSM-5 [9] algorithm [17] had previously been statistically reduced into a 54-item set of “essential” DSM-5 items [13] and then to a 14-item “signposting set” [14], representing the most highly discriminating items from the DISCO DSM-5 algorithm. The 14-item set has high internal consistency (alpha = .92), and ROC curve analyses in an independent validation sample of children with ASD or other clinical conditions (language impairment or intellectual disability) show high sensitivity (.89) and specificity (.89) [14].

For the current study, the 14 signposting interview items [14] were converted into a questionnaire format designed to retain the original concepts that are applied by clinicians in the original interview (e.g. “echolalia” changed to “repeats certain words or phrases out of context”). Descriptions of the 14 items can be found in Additional File 1: Table S2. As coding within the original DISCO interview schedule always corresponded to the most marked behaviour in the context of an autistic difficulty (e.g. “Lack of awareness of others’ feelings”), a subset of five questionnaire items [2, 5, 6, 10, 11] were reversed (e.g. “Aware of others’ feelings”), in line with other questionnaire measures including AQ-10 and SDQ. Parents were asked to give answers based on their child’s behaviour in the last 6 months. The order of questions was identical to the item order in Carrington et al. [14] except for items 13 and 14, which were reversed (Additional file 1: Table S2). A four-point response scale was applied to each statement (Definitely agree, Slightly agree, Slightly disagree, Definitely disagree) to correspond with the AQ-10.

Scoring followed the procedure used for the AQ-10 [11] by converting the four-point scale into binary codes, such that endorsing a behaviour associated with ASD = 1, and endorsing a behaviour *not* associated with ASD = 0. The assignment of binary scores was based on established syntax rules that have been applied to both DSM-IV [15] and DSM-5 [17] DISCO algorithms, as well as the signposting interview items [13, 14]. For seven of the questionnaire items [2, 4, 5, 9–11, 15], the score was dichotomised based on whether participants agreed/disagreed with the statement (e.g. Definitely agree, Slightly agree = 1; Definitely disagree, Slightly disagree = 0). For the other seven items [1, 3, 6–8, 12, 13], only extreme responses were coded as endorsing a behaviour as ASD (e.g. Definitely agree = 1; Slightly agree, Definitely disagree, Slightly disagree = 0). A total score was calculated by adding together scores for each item. This results in an SQ-A total score ranging from 0 to 14, with a higher score reflecting greater endorsement of autistic symptoms. The approach to missing data was the same for all questionnaires: the participant’s mean item score was calculated from the number of items completed and multiplied by the total number of questionnaire items; this was then rounded to the nearest integer to create a pro-rated total score. We allowed for 10% missing data for any single questionnaire.

##### AQ-10 (Child version)

The AQ-10 Child [11] is a shortened version of the 50-item AQ-Child [18]. It has high internal consistency (alpha = .90), high sensitivity (.95) and specificity (.97) [11]. In line with previous research [11, 18], the four-point scale was scored dichotomously, so that autistic traits were marked as absent (0) or present (1). Scores were summed to create a total score out of 10.

##### Strengths and Difficulties Questionnaire (SDQ)

The SDQ (Goodman 2002) is a 25-item parent report measure for 4–16-year-old children that includes subscales related to emotional symptoms, conduct problems, hyperactivity/inattention, peer relationship problems and prosocial behaviour. A review of its psychometric properties across 48 studies reports acceptable internal consistency, modest parent-teacher inter-rater agreement and good discrimination capacity with high sensitivity and specificity [35]. Available syntax (www.sdqinfo.com) was used to calculate the Total Difficulties SDQ score (range 0–40); a higher score indicated greater difficulties.

##### Procedure

Recruitment materials were initially shared with a small number of parents and teachers for feedback, with adjustments made where requested. One hundred and seventy-nine (89.5%) parent participants completed the study online, and the remaining 21 (10.5%) chose to complete a paper version. Welsh translations of the questionnaires were offered, with one participant requesting the Welsh versions. All participating teachers completed it online. The questionnaires were presented in a fixed order (SDQ, SQ-A, AQ-10) followed by the demographic questions. Parents received a small voucher payment and schools received a donation as thanks for participating

#### Statistical analysis

Data were analysed in SPSS 20 [36]. Following the analysis of demographic data and initial data screening, the SQ-A total scores were analysed for reliability and validity. Scale reliability was assessed using Cronbach’s alpha and the mean inter-item correlation (MIIC). Cronbach’s alpha > .70 indicates acceptable internal consistency [37]. For the MIIC, acceptable values fall between .15 and .50 [38]. The questionnaires’ capacity to discriminate the *ASD* group from the *Other* and *None* groups was tested using Kruskal-Wallis and followed up with Mann Whitney tests. The convergent validity of SQ-A in relation to the AQ-10 and discriminant validity in relation to the SDQ was tested by analysing group differences, subgroup differences and correlations between measures. Cross-informant reliability was assessed by looking at parent-teacher correlations for the questionnaires. Bonferroni correction was applied where appropriate, with a conservative threshold of *p* < .01 set for correlations.

## Results

### Demographic data

There were 102 children in the *ASD* group, 52 in the *Other* group and 46 in the *None* group. Of the 102 children with ASD, 77 (75.5%) had one or more co-occurring conditions. See Table 1 for the summary of demographic data. See Additional file 1: Table S1 for the details of reported diagnoses or other conditions provided by parents.

Of the 200 parent/guardian informants, 187 were mothers (93.5%), nine were fathers (4.5%) and two were guardians or other carers (1%). The majority of participants (181; 90.5%) were living in Wales; the remaining 10 who answered the question were based in England or Scotland. Of those who answered questions about nationality and native language, all but one (*N* = 155) identified as British, or British and another European identity, and 144 (93.8%) reported their native language as either English, or bilingual English and Welsh. The majority were employed (*N* = 126; 63%), and the mean age of leaving education was 19.55 years (SD = 3.07).

### Screening of questionnaire data

Little’s Missing Completely at Random (MCAR) tests were carried out on the original, non-recoded items of all the three questionnaires. The tests were non-significant for both the parent and teacher responses, indicating no patterns to missing data. Levels of missing data were generally low (<10%). However, three parents had > 10% missing data on the AQ-10. The patterns of data were not affected by their exclusion, and these participants were retained in the analysis. Six teachers were missing > 10% data on the AQ-10. Further inspection showed the extent of missing data was substantial (mean 42%; range 20–90%), and these participants were excluded from analysis involving the AQ-10.

Distribution for total scores for all parent questionnaires was significantly skewed (Shapiro-Wilk), except for the SQ-A and SDQ in the *ASD* group and the SDQ in the *Other* group. There was one high scoring outlier (± 3 SDs from mean) in the *None* group for the SQ-A in the parent dataset, who was retained in the analysis. For the data included in the parent-teacher reliability analysis, the SQ-A and AQ-10 were both significantly skewed but there were no outliers.

### Questionnaire analysis

#### Descriptive statistics

Every item of the SQ-A was endorsed by at least 30% of the ASD sample. The item percentages for each subgroup (*ASD*, *Other*, *None*) are shown in Additional file 1: Table S2. A Kruskal-Wallis test found no significant age differences between the *ASD*, *Other* and *None* groups (*χ*^2^[2] = 5.18, *p* = .075). Table 2 shows the descriptive results for the three questionnaires as well as relevant correlations.

**Table 2:** Parent-report questionnaire data for the UK sample

	Whole sample (*N* = 200)	ASD(*N* = 102)	Other(*N* = 52)	None(*N* = 46)
SQ-A				
*Mean (SD)*	5.14 (3.91)	7.39 (3.19)	4.08 (3.37)	1.33 (2.06)
*Range*	0–14	0–14	0–12	0–9
*Median (IQR)* *Cronbach’s α* *MIIC*	5 (6) .87 .32	7 (5) .76 .18	3 (6) .83 .25	0 (2) .78 .20
*Correlation with AQ-10 and SDQ (Spearman’s r*_s_*)*				
AQ-10 SDQ	.79*** .65***	.46*** .19	.76*** .71***	.65*** .55***
AQ-10				
*Mean (SD)*	6.39 (3.19)	8.41 (1.37)	5.71 (3.1)	2.65 (2.41)
*Range*	0–10	5–10	0–10	0–9
*Median (IQR)*	7 (5)	9 (2)	6 (6)	2 (3)
SDQ				
*Mean (SD)*	19.49 (7.31)	22.92 (5.03)	18.87 (7.33)	12.57 (6.51)
*Range*	3–37	11–37	4–31	3–29
*Median (IQR)*	20 (10)	23 (7)	18.5 (13)	11.5 (9)
*Correlation between AQ-10 and SDQ*				
*Spearman’s r*_s_	.63***	.16	.66***	.53***

*ASD* autism spectrum disorder, *AQ-10* Autism Spectrum Quotient-10-Child, *MIIC* mean inter-item correlation, *SDQ* Strengths and Difficulties Questionnaire, *SQ-A* Signposting Questionnaire for Autism

***p < .001

#### Reliability and validity of the SQ-A

*Scale reliability*: Cronbach’s alpha and MIIC showed acceptable to good internal consistency for every group (see Table 2).

*Clinical diagnosis of ASD*: A Kruskal-Wallis test showed significant differences between the *ASD*, *Other* and *None* in SQ-A scores (*χ*^2^[2] = 86.31, *p* < .001). Follow-up Mann-Whitney tests with a Bonferroni correction (.05/9 = .006) found the *ASD* group scored significantly higher than both the *Other* (*Z* = − 5.34, *p* < .001) and *None* (*Z* = − 8.62, *p* < .001) groups, and in turn the *Other* group scored significantly higher than the *None* group (*Z* = − 4.53, *p* < .001).

*Convergent validity*: *AQ-10*. Following a significant Kruskal-Wallis test between groups (*χ*^2^[2] = 92.58, *p* < .001), the AQ-10 showed an identical pattern of group difference to the SQ-A; *ASD* vs *Other* (*Z* = − 5.29, *p* < .001; *ASD* vs *None* (*Z* = − 9.07, *p* < .001); *Other* vs *None* (*Z* = − 4.69, *p* < .001). The SQ-A scores significantly correlated with the AQ-10 scores, for the whole sample and every diagnostic group (see Table 2).

*Discriminant validity: SDQ*. Following a significant Kruskal-Wallis test between groups (*χ*^2^[2] = 57.46, *p* < .001), the SDQ showed an identical pattern of group difference to the other questionnaires: *ASD* vs *Other* (*Z* = − 3.05, *p* = .002), *ASD* vs *None* (*Z* = − 7.53, *p* < .001) and *Other* vs *None* (*Z* = − 4.09, *p* < .001). The SQ-A scores significantly correlated with the SDQ scores for the whole sample and the *Other* and *None* groups but not for the *ASD* group (Table 2). Similarly, the correlations between SDQ and AQ-10 scores were significant for all groups except for the ASD group (Table 2).

*Comparison between ASD-only and ASD-co-occurring subgroups*: To examine the effect of co-occurring conditions, further analysis separated the *ASD* group into *ASD-only* (ASD and no other reported conditions, *N* = 25) and *ASD-co-occurring* group (*N* = 77; see Additional file 1: Table S3) and compared scores with the *Other* group (*N* = 52; see Table 2). Kruskal-Wallis tests indicated a difference between groups for the SDQ (*χ*^2^[2] = 15.94, *p* < .001), SQ-A (*χ*^2^[2] = 30.67, *p* < .001) and AQ-10 (*χ*^2^[2] = 30.3, *p* < .001). Follow-up Mann-Whitney tests were applied, with a Bonferroni correction of .006. First, the SDQ was higher for the *ASD-co-occurring* group than that for the *ASD-only* group (*Z* = − 2.88; *p* < .004), but there was no significant difference between these subgroups for the SQ-A (*Z* = − 1.61, *p* = .12) or AQ-10; (*Z* = − 1.76; *p* < .08). Second, comparisons between these subgroups and the *Other* group showed that the SDQ was higher in the *ASD-co-occurring* group than in the *Other* group (*Z* = − 3.52; *p* < .001) but did not distinguish the *ASD-only* group from the *Other* group (*Z* = − .71; *p* = .48). In contrast, the SQ-A and AQ-10 were both higher in the *ASD-only* versus *Other* group (SQ-A; *Z* = − 2.86, *p* = .004; AQ-10; *Z* = 2.93; *p* = .003), as well as higher in the *ASD-co-occurring* versus *Other* group (SQ-A; *Z* = − 5.45, *p* < .001; AQ-10; *Z* = − 5.36; *p* < .001).

*Cross-informant reliability*: Teacher-completed questionnaires were provided for a subset of 38 children (27 male). Of these, 20 were from the *ASD* group, 15 from the *Other* and 3 from the *None*; all groups were analysed together. The reliability for the teacher-reported SQ-A was good (*α* = .86). Parents and teachers were significantly correlated for the SQ-A (*r*_s_ = .6, *p* < .001) although teachers rated children significantly lower than parents (Parent *M* = 5.26 (SD = 4.42); Teacher *M* = 3.97 (SD = 3.57); *Z* = − 2.15, *p* = .03). Parent and teacher reports were also significantly correlated for AQ-10 (*r*_s_ = .61, *p* < .001) and for SDQ (*r*_s_ = .34, *p* < .04). Teachers also rated children significantly lower on the SDQ (Parent *M* = 18.55 (SD = 6.36); Teacher *M* = 13.21 (SD = 6.58); *Z* = − 3.72, *p* < .001), but not on the AQ-10 (Parent *M* = 5.79 (SD = 3.06); Teacher *M* = 5.42 (SD = 3.21); *Z* = − .53, *p* = .53).

## Study2—children from Latvia

### Participants

Participants were 110 parents or guardians of children aged between 4 and 11 years, from Riga, Latvia, and the surrounding area. The parents were recruited from two special education schools and three mainstream schools in Riga, and one mainstream school with inclusive education in the Riga region. Parents of autistic children were additionally recruited via social media, targeting parents/guardians aged 25–50 years in Riga and the surrounding region. Finally, three centres for autism assessment, intervention and consultations supported recruitment. Two participants had missing data on more than 10% of the three questionnaires, and four children were outside the target age range of 4–11 years old. Therefore, the final sample was *N* = 104. Teacher data were not included in this study.

### Materials

Parents were asked the same demographic questions as in study 1, but the following questions were not included: participant nationality, location and native language. The question about SEN status (not applicable in Latvia) was substituted with a question about whether the child received additional support for educational, emotional or behavioural needs. The same three questionnaires were used and scored in the same way as study 1. The Latvian version of the SDQ was used in the current study. Permission for translation of the AQ-10 was obtained from the Autism Research Centre, Cambridge, UK. The SQ-A and AQ-10 were translated by a professional translation organisation and then revised, including checking for English back translation, by IB and another expert in clinical and developmental psychology.

### Procedure

Seventy participants completed the study online, and 40 completed paper versions of the questionnaires. The procedure was identical to study 1.

### Statistical analyses

The data were analysed in the same way as study 1. The high percentage of children with co-occurring conditions meant that it was not possible to compare *ASD-only* and *ASD-co-occurring* subgroups. Additional analyses were conducted to test for statistical differences in questionnaire scores between countries.

### Results

#### Demographic data

There were 35 children in the *ASD* group, 40 in the *Other* group and 29 in the *None* group. Of the 35 children with ASD, parents reported that 33 (94.3%) had one or more co-occurring conditions. Eighteen parents of children in the *Other* group reported that they thought their child might have ASD, and nine of these were seeking a diagnosis. Details of reported diagnoses or other conditions/impairments for the whole sample, and the *ASD* and *Other* groups, are shown in Additional file 1: Table S4. Of the 104 parent/guardian informants, 102 were mothers (97.1%) and the majority of informants were currently employed (75.7%). Seventy-six participants disclosed the age they left education, with a mean of 24.59 years (SD = 5.6). Demographic data are shown in Table 3.

**Table 3:** Demographic data for the whole Latvia sample and split by *ASD*, *Other* and *None* groups

	Total (*N* = 104)	ASD (*N* = 35)	Other (*N* = 40)	None (*N* = 29)
Age (years)§	8.11 (1.83)	7.97 (1.81)	7.88 (1.91)	8.71 (1.65)§
Gender	61 (62.9%) M 36 (37.1%) F	30 (90.9%) M‡ 3 (8.8%) F	24 (60%) M 16 (40%) F	7 (29.2%) M§ 17 (70.8%) F
Language expression				
None	4 (3.9%)†	3 (8.6%)	1 (2.6%)†	0 (0%)
Single words	8 (7.8%)	7 (20%)	1 (2.6%)	0 (0%)
Simple phrases	14 (13.6%)	8 (22.9%)	6 (15.4%)	0 (0%)
Full sentences	77 (74.8%)	17 (48.6%)	31 (79.5%)	29 (100%)
Language comprehension				
None	2 (1.9%)	0 (0%)	2 (5%)	0 (0%)
Single words	2 (1.9%)	2 (5.7%)	0 (0%)	0 (0%)
Simple phrases	14 (13.5%)	11 (31.4%)	3 (7.5%)	0 (0%)
Full sentences	86 (82.7%)	22 (62.9%)	35 (87.5%)	29 (100%)

Standard deviation (SD) reported in brackets for age; percentage reported in brackets for gender, language use and language comprehension

*ASD* autism spectrum disorder

†Missing = 1

‡Missing = 2

§Missing = 5

¶Missing = 7

#### Screening of questionnaire data

Little’s MCAR tests were non-significant for all the three questionnaires. Levels of missing data were generally low; however, one participant had > 10% missing data on the SQ-A, and three participants had > 20% missing data on the AQ-10; removing these participants did not affect the pattern of results, and so, they remained in the analyses. There were two participants with no data on the SDQ, who were excluded from analyses that include this variable.

### Questionnaire analysis

#### Descriptive statistics

Item frequency percentages per diagnostic group are shown in Additional file 1: Table S2 next to the endorsements for the UK sample. SQ-A items were endorsed for between 14.7 and 82.9% of the *ASD* group, except for question 6 (emotionally expressive gestures), which was endorsed for only 5.7%.

The distributions of the SQ-A and AQ-10 scores were significantly skewed (Shapiro-Wilk), with the exception of the SQ-A scores in the *ASD* group. There was one outlier in the *ASD* group on the AQ-10 (> 3 SDs below the mean) and one outlier in the *None* group on the SQ-A and AQ-10 (> 3 SDs above the mean); both were kept in the analysis. A Kruskal-Wallis test showed no differences between the *ASD*, *Other* and *None* groups for age (*χ*^2^[2] = 3.29, *p* = .19).

#### Reliability and validity of the SQ-A

*Scale reliability*: Cronbach’s alpha and MIIC showed acceptable internal consistency across the whole sample and in the *ASD* group and less than acceptable internal consistency in the *Other* and *None* groups (Table 4).

**Table 4:** Parent-report questionnaire data for the Latvia sample

	Whole sample (*N* = 104)	ASD (*N* = 35)	Other (*N* = 40)	None (*N* = 29)
SQ-A				
*Mean (SD)*	3.36 (2.99)	5.74 (3.09)	2.88 (2.12)	1.1 (1.37)
*Range*	0–12	1–12	0–8	0–6
*Median (IQR)*	3 (4)	6 (5)	3 (4)	1 (2)
*Cronbach’s*	.79	.77	.56	.61‡
*MIIC*	.2	.19	.06	.12
*Correlation with AQ-10 and SDQ (Spearman’s r*_s_*)*				
AQ-10 SDQ	.72*** .59***	.50** .41^+^	.55*** .53**	.35 .02
AQ-10				
*Mean (SD)*	5.1 (3.65)	8.17 (2.3)	4.83 (3.28)	1.72 (2.05)
*Range*	0–10	1–10	0–10	0–8
*Median (IQR)*	5 (8)	9 (3)	4.5 (6)	1 (3)
SDQ				
*Mean (SD)*	17.4 (7.55)	21.8 (5.68)	18.36 (6.68)†	10.57 (5.92)†
*Range*	1–33	9–33	4–28	1–22
*Median (IQR)*	18 (12)	22 (7)	19 (8)	9.5 (9)
*Correlation between AQ-10 and SDQ*				
*Spearman’s r*_*s*_	.67***	.28	.63***	.31

*ASD* autism spectrum disorder, *AQ-10* Autism Spectrum Quotient-10-Child, *MIIC* mean inter-item correlation, *SDQ* Strengths and Difficulties Questionnaire, *SQ-A* Signposting Questionnaire for Autism

***p < .001; **p < .01; ^+^*p* = .014 (above corrected threshold *p* < .01)

†Missing = 1

‡Four items (2, 3, 7 and 13) had zero variance and were therefore removed from the reliability analysis

*Clinical diagnosis of ASD*. A Kruskal-Wallis test showed significant differences for the SQ-A (*χ*^2^[2] = 40.75, *p* < .001). Follow-up Mann-Whitney tests were run with a Bonferroni correction (= .05/9 = .006). The *ASD* group scored significantly higher than both the *Other* (*Z* = − 3.95, *p* < .001) and *None* (*Z* = − 5.86, *p* < .001) groups, and the *Other* group scored significantly higher than the *None* group (*Z* = − 3.6, *p* < .001).

*Convergent validity*: *AQ-10*. As for study 1, the AQ-10 showed an identical pattern of group difference to the SQ-A; (*χ*^2^[2] = 49.42, *p* < .001; *ASD* vs. *Other*: *Z* = − 4.39, *p* < .001; *ASD* vs. *None*: *Z* = − 5.86, *p* < .001; *Other* vs. *None*: *Z* = − 3.6, *p* < .001). The SQ-A also correlated strongly with the AQ-10 for the whole sample, the *ASD* and *Other* groups, but unlike study 1 did not reach significance (*p* = .06) in the *None* group.

*Discriminant validity*: *SDQ*. Following a significant main group effect for the SDQ (*χ*^2^[2] = 40.75, *p* < .001), both the *ASD* and *Other* groups scored higher on the SDQ than the *None* group (*ASD* vs. *None*: *Z* = − 5.49, *p* < .001; *Other* vs. *None*: *Z* = − 4.24, *p* < .001). However, the difference between the *ASD* and *Other* groups was not significant at the Bonferroni corrected level (*Z* = − 2.02, *p* = .04). Like study 1, the SQ-A correlated strongly with the SDQ for the whole sample and the *Other* group but did not reach the *p* < .01 threshold for the *ASD* group. Unlike study 1, correlations were also not significant in the None group. The AQ-10 also correlated significantly at *p* < .01 with the SDQ in the whole sample and the *Other* group, but did not reach corrected or uncorrected significance thresholds in the *ASD* or *None* groups.

#### Comparison between the UK and Latvian data

The Latvian group were slightly older. This difference was not significant at the whole group level, and not significant for any individual group (Bonferroni correction: .05/3 = .017). For the number of children speaking or understanding full sentences, there were no significant differences in language at the whole group level, or for any specific group. At the whole group level, UK parents produced higher scores for all the three questionnaires (SQ-A: *Z* = − 3.65, *p* < .001; AQ-10: *Z* = − 2.74, *p* < .006; SDQ: *Z* = − 2.21, *p* = .027). Further investigation found that there were no significant differences when the data were analysed by group for the *Other* or *None* groups. For the *ASD* group, the Latvian parents gave lower scores on all questionnaires and significantly lower scores on the SQ-A (*Z* = − 2.501, *p* = .012), although this result does not survive Bonferroni correction (= .05/9 = .006).

## Discussion

A new autism signposting questionnaire (SQ-A) was developed that, used published DSM-5 items from a clinical interview [13, 14]. The items in the SQ-A had previously been identified as the 14 most discriminating items in the DISCO DSM-5 algorithm set [13, 14]. We found that total mean scores for 4–11-year-old autistic children were significantly higher than non-autistic children, including those with a range of other clinical conditions reported by parents. Our findings provide preliminary evidence of the utility of the 14-item set in a parent-report and teacher-report questionnaire format. Importantly, these findings applied not only to the UK but also to Latvia, a country with fledgling diagnostic services and limited national initiatives to support autism awareness. These findings have both clinical and scientific implications for autism research.

Our primary goal was to develop a signposting questionnaire based on the latest diagnostic criteria for ASD and to co-ordinate with DSM-5 compatible assessments, such as the DISCO. Importantly, this is the first measure of its type to be based on DSM-5 items. By working with a set of interview items from the DISCO that had already been published with a full sensitivity and specificity analysis [14], we were able to explore whether this set of items could be effectively answered by parents. Our approach involved comparing performance of the SQ-A with the child version of the AQ-10, an existing short autism questionnaire, and the SDQ, a more general measure of childhood difficulties. Autistic children had higher scores on both the SQ-A and the AQ-10 relative to children with a range of other conditions (*Other* group) and children without any known difficulties (*None* group). In particular, the inclusion of the *Other* group provided a stringent measure of specificity. The two measures also had high convergent validity, despite differences in the questions. For example, 11 of the 14 items in the SQ-A (78.57%) related to social communication behaviours, with seven (63.64%) relating to the socio-emotional reciprocity subdomain and four relating to deficits in developing and maintaining relationships (36.36%). In the AQ-10, seven (70%) items could be considered social-communication items [3, 5–10], with three (42.86%; 3, 5, 6) relating to socio-emotional reciprocity and four (57.14%; 7, 8, 9, 10) relating to deficits in developing and maintaining relationships. Moreover, only the AQ-10 included an item relating to sensory symptoms and had a greater emphasis on cognitive processes. The establishment of convergent validity holds promise for future analyses of symptoms of neurodevelopmental disorders and permits the study of the latent construct of autism-relevant symptoms in multi-trait, multi-method designs [25, 39–41]. Multivariate analyses that employ more than one questionnaire to estimate latent constructs convey additional predictive power. Additionally, approaches that use multiple questionnaires enable the latent effect of respondent to be modelled, which can be particularly important when considering correlations between different types of psychopathology or behavioural traits [42].

In contrast to the autism-specific measures, the SDQ performed differently when children with a singular diagnosis of ASD were compared to those with at least one other co-occurring condition. For the SDQ, scores were higher in the co-occurring group than the *ASD-only* group. Further, the *ASD-only* group did not perform significantly differently to the group with a range of other conditions on the SDQ. These data confirm previous reports that the SDQ performs differently when autism co-occurs with other conditions (e.g. [27]). In contrast, the SQ-A and AQ-10 targeted autistic behaviours specifically and scores were not affected by the presence of other conditions. The pattern of findings, particularly the absence of a significant correlation between the SQ-A and SDQ for the ASD group in both countries, confirmed the discriminant validity of the SQ-A. An additional analysis established cross-informant reliability for the SQ-A, AQ-10 and SDQ, although teachers gave significantly lower scores than parents on the SQ-A and SDQ. However, the sample size was small and it was not possible to investigate group differences.

A key contribution of the SQ-A is to provide frontline professionals and parents with an accessible and short questionnaire that focuses on the pattern of a child’s behaviour across the 14 most discriminating items from a clinical interview, the DISCO [14]. Although we calculated a total score in the current study to enable comparison across groups, the SQ-A could hold value simply as a checklist (using the 0/1 scoring outlined in the Method) to orient professionals to key behaviours. Even if a child who is struggling at school does not have a diagnosis, the SQ-A can help teachers recognise and understand whether there is a pattern of behaviours in their pupils that may affect their functioning. This current use of the tool follows the original ethos of the DISCO, with its emphasis on interpreting a pattern of behaviours rather than providing a quantitative measure of categorisation [16]. In this context, the SQ-A could work alongside government resources to help focus teachers and other frontline professionals on the key signs of autism and their varied presentation, facilitating the implementation of appropriate support (see the Welsh Local Government Association initiatives at www.ASDInfoWales.co.uk and www.autismchildsigns.com for an example).

In the longer term, the newly developed SQ-A has the potential to provide a time-saving contribution to the lengthy and complex process of DSM-5 diagnosis for primary schoolchildren and their families (see [2, 43]). However, additional research is needed to develop both the SQ-A and AQ-10 questionnaires before they singularly inform the diagnostic referral process. For the SQ-A, analysis of sensitivity and specificity would be required as well as a prospective research design with a population referred by frontline professionals with a range of developmental concerns. Further examination of its measurement characteristics is also needed to understand the lower relative scores compared to the AQ-10 as well as the lower scoring by teachers compared to parent report. In the meantime, the clear diagnostic group differences established in the current study, along with convergence with the AQ-10, mean that the questionnaire could help give frontline professionals confidence in raising concerns. Notably, it should be remembered that signposting questionnaires provide a single contribution to clinical practice and education settings and are not a shortcut replacement for best-practice diagnostic procedures.

Our second goal was to investigate the performance of the SQ-A in Latvia, a country with more limited autism awareness and provision. Reliability and validity of the SQ-A was also demonstrated in this country, where it performed similarly to the AQ-10. Transforming the DISCO items into a questionnaire format is particularly beneficial to countries like Latvia, where services are less developed. Latvian professionals can have the advantage of accessing signs of autism that are based on concepts drawn from the DISCO DSM-5 algorithm [15, 16], even if access to a clinical interview is not possible. The more limited availability of diagnostic services also makes it particularly important that accessible tools for frontline professionals are available. Identifying signs, and particularly the pattern of signs, can aid frontline professionals in supporting children, regardless of their diagnostic status.

It was important to explore the questionnaire in Latvia as parents from countries with more limited understanding of ASD may respond differently. It is notable that the Latvian parents tended to give lower scores across all the three questionnaires. This may reflect less robust awareness and understanding of autism by Latvian parents or an oversensitivity to possible autism signs by UK parents. However, conclusions are limited by the lack of objective measures of autism symptomatology between countries. A broader possibility is the effect of culture on parents’ recognition and interpretation of autistic behaviours [44]. For example, the importance of social relatedness between mother and child in India has been argued to drive why social difficulties are commonly first noticed by Indian parents [45]. In contrast, emphasis on language development milestones among American parents may explain why they initially noticed delayed language development over social communicative difficulties [46]. Although item-analysis was beyond the scope of this study, there were some individual questionnaire items where the two countries appeared discrepant. For example, only 5.9% of Latvian parents thought their autistic child showed a lack of emotionally expressive gestures compared to a third of UK parents. Whether this reflects culturally different norms for levels of emotional expression or a broader conceptualisation of expressive gestures (e.g. crying) in Latvia would require further investigation. This latter point also relates to linguistic variation and the possibility of question ambiguity in the translated questionnaire [10]. A recent cross-cultural analysis of the 50-item AQ found that although there were five items that were excellent predictors of autism in India, Japan and the UK, the majority of items that were excellent predictors only performed well in one or two countries, and four items showed clear evidence of cultural differences in responding [10]. However, despite item-level cultural variation, overall, the AQ had excellent sensitivity and specificity for all three countries. The current results indicate the potential of the SQ-A and AQ-10 to inform autism referral in countries with different cultural norms to the UK and more limited public and professional awareness of autism. Further research is needed with the SQ-A to explore differences in responses between countries, both at the level of single items and in terms of the pattern of items.

Our final goal was to provide further psychometric information about two established questionnaires, the AQ-10 and SDQ. As far as we are aware, this is the first study to test the performance of the AQ-10 Child as a stand-alone questionnaire, rather than as a post hoc extraction of the 10 items from the full AQ, overcoming previous issues with item context effects [47]. It is also the first study to show that the AQ-10 can discriminate between autistic children and a comparison group with a range of SEN, as well as establishing cross-informant reliability. For the SDQ, the analysis of ASD subgroups highlighted the importance of attending to co-occurrence when interpreting SDQ scores in autistic children. As with the other measures, parent and teacher scores were significantly correlated, although the teachers gave significantly lower scores. Further testing should now be carried out comparing the SQ-A and AQ-10 with other autism-related questionnaires such as the SCDC [20]. Given the strong association between SCDC and SDQ [48], which has been difficult to disentangle [49], further research comparing subgroupings with and without co-occurring conditions could help to delineate the social and communication behaviour independently of other emotional and behavioural difficulties.

An important final point is to highlight the value of measurement development within the autism scientific community. The changing conceptualisation of autism requires a dynamic clinical and research landscape in which measures are compared, contrasted, and adapted over time. The use of multiple assessments supports the application of more sophisticated analyses, where autism can be captured by the shared variance across measures. New measures, such as the SQ-A, can therefore only contribute positively to the understanding of existing measures as well as provide more choice to researchers and clinical and educational professionals. For example, there was high convergent validity between the SQ-A and AQ-10 despite different types of questions, with a greater emphasis on cognitive and social-cognitive processes in the AQ-10 and a bias towards observed behaviours in the SQ-A. The ultimate goal of this research is therefore to promote better understanding and provide more scope within clinical and educational practice to help families.

## Limitations

Limitations of the research included the fact that independent verification of diagnoses was not possible. Although there is good evidence of the reliability of parent-report of diagnosis in this context [50], parents’ and teachers’ knowledge of diagnosis may have biased endorsement of a known autistic feature. Another limitation was the high proportion of male participants in both countries as well as in the original signposting analysis [14]. Issues such as better camouflaging of autistic signs and poorer recognition of autism in females [51] suggest that questionnaires that identify autism features should be assessed for sex biases [52]. Another important limitation is that there is need for stakeholders to systematically assess the usability and acceptability of items within the SQ-A questionnaire, including their appropriateness in different countries.

Although the SQ-A is strongly research evidence-based, it was conceptually derived by applying statistical reduction to a large number of reliable and valid items [13], following an established procedure [21]. This is distinct from an empirically derived, factor analytic approach, which is an alternative way of reducing items. While not a limitation per se, the genesis of the SQ-A is important to consider when evaluating and understanding its scope. The questionnaire documents patterns of behaviour in line with DSM-5; however, the measure does not include all the DSM-5 subdomains, which limits its potential use for referral. This reflects the composition of items in the DISCO. For example, the DISCO includes many sensory items, which focus on different types of sensory behaviours and experiences (e.g. indifference to heat and cold, distress caused by sounds, unusual interest in the feel of surfaces). Although there is high endorsement by autistic people of sensory items, the pattern of endorsement varies by individual [53]. This means that no single sensory item was likely to be one of the most discriminating and was therefore not derived during statistical reduction. Further research could explore the extent to which the pattern of responding on the 14 items relates to scores in excluded subdomains.

The scope of the research, which included a relatively small sample size and unconfirmed diagnoses, did not permit an analysis of the sensitivity and specificity of the SQ-A as a tool for identifying ASD, as is common with other measures, including the AQ-10. The sensitivity and specificity of the 14-item set in an interview format has been established, as this item set formed part of a series of studies examining the diagnostic algorithms [14]. At this stage of development, however, it would be premature to support the use of the SQ-A in clinical practice as a categorical indicator of ASD. Nevertheless, the SQ-A is still valuable in its current form, by making available for professionals and parents a simple set of DSM-5-related signs that provide useful information about a child’s profile of autistic behaviours. To complete its development, however, consultation from the autistic community is needed and further research is required with different samples, including those that have been clinically referred. Finally, more in-depth exploration of how the SQ-A works alongside complementary and more in-depth tools is necessary.

## Conclusions

In conclusion, measurement comparison of the 14-item parent SQ-A, the AQ-10 and the SDQ in the UK and Latvia indicate that all the three questionnaires have sound psychometric characteristics. They have potential to usefully support the ASD referral and diagnostic process for children, whether or not there are well-established services and long-standing government strategies for professional and public awareness. We encourage parents and professionals to consider the distinct pattern of different individual behaviours that are identified when using the SQ-A while bearing in mind the limitations of its current scope and purpose. We also encourage further research into this pattern of key indicators, as well as other autism signs, with larger samples and across other countries.

## Supplementary information

**Additional file 1: Table S1.** Frequencies of parents reporting diagnoses other than autism spectrum disorder for the whole UK sample, and for the *ASD* and *Other* groups. **Table S2.** Parent report percentage endorsement of each Signposting Questionnaire for Autism (SQ-A) item shown by country and diagnostic group. **Table S3.** Group differences between *ASD-only* and *ASD-co-occurring* subgroups for the three parent-report questionnaires in the UK sample. **Table S4.** Frequencies of parents reporting diagnoses other than autism spectrum disorder for the whole Latvia sample and for the *ASD* and *Other* groups

## Data Availability

The datasets used and/or analysed during the current study are available from the corresponding author on reasonable request. The SQ-A will be available from the corresponding author on reasonable request.

## Abbreviations

ADI-R: Autism Diagnostic Interview-Revised
ADOS: Autism Diagnostic Observation Schedule
ASD: Autism spectrum disorder
AQ-Child: Autism Spectrum Quotient-Child version
AQ-10: Short Autism Spectrum Quotient-Child version
DISCO: Diagnostic Interview for Social and Communication Disorders
DSM-IV: Diagnostic and Statistical Manual of Mental Disorders–fourth edition
DSM-5: Diagnostic and Statistical Manual of Mental Disorders–fifth edition
MCAR: Little’s Missing Completely at Random
MIIC: Mean inter-item correlation
ROC: Receiver operating characteristic
SQ-A: Signposting Questionnaire for Autism
SDQ: Strengths and Difficulties Questionnaire

## Footnotes

1 Note that we use the term autism spectrum disorder (ASD) when referring to the current clinical label used for autism diagnosis. Elsewhere we use the ‘identity-first’ language of autistic person, or the term autism (see 1. Kenny L, Hattersley C, Molins B, Buckley C, Povey C, Pellicano E. Which terms should be used to describe autism? Perspectives from the UK autism community. Autism. 2016;20(4):442-62.)
