# Supplementary material for: Development of the Signposting Questionnaire for Autism (SQ-A): measurement comparison with the 10-item Autism Spectrum Quotient-Child and the Strengths and Difficulties Questionnaire in the UK and Latvia
Source: Mol Autism. 2020 Aug 15;11:64. doi: 10.1186/s13229-020-00368-9 (PMC7429457; doi:10.1186/s13229-020-00368-9)
Supplement: Supplementary file 1 — Additional file 1: Table S1. Frequencies of parents reporting diagnoses other than autism spectrum disorder for the whole UK sample, and for the ASD and Other groups. Table S2. Parent report percentage endorsement of each Signposting Questionnaire for Autism (SQ-A) item shown by country and diagnostic group. Table S3. Group differences between ASD-only and ASD-co-occurring subgroups for the three parent-report questionnaires in the UK sample. Table S4. Frequencies of parents reporting diagnoses other than autism spectrum disorder for the whole Latvia sample and for the ASD and Other groups [file 13229_2020_368_MOESM1_ESM.docx]

**Additional File 1**

**Table S1:** Frequencies of parents reporting diagnoses other than autism spectrum disorder for the whole UK sample, and for the *ASD* and *Other* groups.

| **Diagnosis or condition** | **Whole sample (*N*=200)** | **ASD (*N*=102)** | **Other (*N*=52)** |
| --- | --- | --- | --- |
| Speech or language impairment | 50 (25%) | 33 (32.4%) | 17 (32.7%) |
| ADHD | 25 (12.5%) | 21 (20.6%) | 4 (7.7%) |
| DCD | 20 (10%) | 16 (15.7%) | 4 (7.7%) |
| CD or ODD | 3 (1.5%) | 3 (2.9%) | 0 (0%) |
| Anxiety Disorder | 17 (8.5%) | 11 (10.8%) | 6 (11.5%) |
| Tourette’s Syndrome | 2 (1%) | 2 (2%) | 0 (0%) |
| Genetic disorder | 18 (9%) | 11 (10.8%) | 7 (13.5%) |
| Hearing impairment | 11 (5.5%) | 4 (3.9%) | 7 (13.5%) |
| Visual impairment | 19 (9.5%)† | 10 (9.8%) | 8 (15.4%) |
| Intellectual or learning disability | 63 (31.5%) | 44 (43.1%) | 19 (36.5%) |
| Physical disability | 10 (5%) | 6 (5.9%) | 4 (7.7%) |

ADHD = Attention deficit/hyperactivity disorder; ASD = autism spectrum disorder; DCD = Developmental coordination disorder; CD = Conduct disorder; ODD = Oppositional defiant disorder. †One participant that a parent identified as having a visual impairment was put in the *None* group as the child had corrected vision (glasses).

**Table S2:** Parent report percentage endorsement of each Signposting Questionnaire for Autism (SQ-A) item shown by country and diagnostic group. Note that column 1 (content of DISCO item) provides content descriptors and not the full SQ-A questions (with reversals) for clarity of presentation.

|  | **UK sample**‡  (*N*=200) | | | **Latvia sample**§  (*N*=104) | | |
| --- | --- | --- | --- | --- | --- | --- |
| **Content of DISCO item**† | **ASD**  (*N*=102) | **Other**  (*N*=52) | **None**  (*N*=46) | **ASD**  (*N*=35) | **Other**  (*N*=40) | **None**  (*N*=29) |
| 1. Makes one-sided approaches | 67.6 | 34.6 | 8.7 | 17.1 | 15.0 | 10.3 |
| 2. Does not seek comfort when in pain or distress | 30.4 | 7.7 | 6.5 | 34.3 | 17.5 | 0 |
| 3. Does not offer comfort to others | 34.3 | 11.5 | 2.2 | 31.4 | 7.5 | 0 |
| 4. No interest in age peers | 55.4 | 36.5 | 10.9 | 48.6 | 17.5 | 3.4 |
| 5. Sharing interests limited or absent | 62.7 | 44.2 | 17.4 | 45.7 | 22.5 | 10.7 |
| 6. Lack of emotionally expressive gestures | 33.3 | 11.5 | 2.2 | 5.9 | 5.0 | 3.6 |
| 7. No emotional response to age peers | 34.3 | 7.7 | 4.3 | 25.7 | 7.5 | 0 |
| 8. Lack of joint reference pointing | 29.4 | 13.5 | 4.3 | 14.7 | 10.00 | 17.2 |
| 9. Lack of friendship with age peers | 60.8 | 41.2 | 2.2 | 65.7 | 42.5 | 10.3 |
| 10. Does not interact with peers | 62.7 | 44.2 | 19.6 | 62.9 | 35.0 | 41.4 |
| 11. Lack of awareness of others’ feelings | 63.7 | 32.7 | 13.0 | 65.7 | 37.5 | 6.9 |
| 12. Delayed echolalia | 58.0 | 36.6 | 8.7 | 40.0 | 15.0 | 3.4 |
| 13. Arranges objects in patterns | 53.9 | 25.0 | 10.9 | 29.4 | 25.0 | 0 |
| 14. Limited pattern of self-chosen activities | 92.2 | 61.5 | 21.7 | 82.9 | 30.0 | 3.4 |

ASD = autism spectrum disorder; DISCO = Diagnostic Interview for Social and Communication Disorders; †Items listed are the item headers reproduced from Carrington et al., (2015). These headers were adapted for use in the SQ-A, which is not reproduced fully here. ‡Missing data for 4 UK participants; 1 participant missing for Q4 and 2 missing for Q12 (ASD) and 1 for Q9 (Other). §Missing data for 4 Latvian participants: 1 participant missing for Q8 and Q13 (ASD), 1 missing for Q5 (None), 2 missing for Q6 (ASD and None). Table accounts for missing data by showing valid percent.

**Table S3**: Group differences between *ASD-only* and *ASD-co-occurring* subgroups for the three parent-report questionnaires in the UK sample.

|  | **ASD-only**  **N=25** | | **ASD-co-occurring**  **N=77** | **Other**  **(N=52)** |
| --- | --- | --- | --- | --- |
| **SQ-A** | |  |  |  |
| *Mean (SD)* | 6.48 (3.33) | | 7.69 (3.11) | 4.08 (3.37) |
| *Median (IQR)* | 6.00 (5.00) | | 8.00 (5.00) | 3.00 (5.75) |
| **AQ-10** |  | |  |  |
| *Mean (SD)* | 7.96 (1.54) | | 8.56 (1.28) | 5.71 (3.1) |
| *Median (IQR)* | 8.00 (2.50) | | 9.00 (2.00) | 6.00 (5.75) |
| **SDQ** |  | |  |  |
| *Mean (SD)* | 20.4 (4.05) | | 23.74 (5.07) | 18.87 (7.33) |
| *Median (IQR)* | 21.00 (5.50) | | 24.00 (7.00) | 18.50 (12.75) |

ASD = autism spectrum disorder; AQ-10 = Autism Spectrum Quotient-10 - Child; SDQ = Strengths and Difficulties Questionnaire; SQ-A = Signposting Questionnaire for Autism.

**Table S4:** Frequencies of parents reporting diagnoses other than autism spectrum disorder for the whole Latvia sample and for the *ASD* and *Other* groups.

| **Diagnosis or condition** | **Whole sample (*N*=104)** | **ASD**  **(*N*=35)** | **Other**  **(*N*=40)** |
| --- | --- | --- | --- |
| Speech or language impairment | 41 (39.4%) | 27 (77.1%) | 14 (35%) |
| ADHD | 19 (18.3%) | 9 (25.7%) | 10 (25%) |
| DCD | 10 (9.6%) | 6 (17.1%) | 4 (10%) |
| CD or ODD | 22 (21.2%) | 14 (40%) | 8 (20%) |
| Anxiety disorder | 6 (5.8%) | 4 (11.4%) | 2 (5%) |
| Tourette’s Syndrome | 0 (0%) | 0 (0%) | 0 (0%) |
| Genetic disorder | 6 (5.8%) | 4 (11.4%) | 2 (5%) |
| Hearing impairment | 2 (1.9%) | 2 (5.7%) | 0 (0%) |
| Visual impairment† | 14 (13.3%) | 5 (14.3%) | 9 (22.5%) |
| Mental health problem or learning disability† | 29 (28.4%) | 19 (54.3%) | 10 (25%) |
| Physical disability‡ | 6 (5.9%) | 3 (8.6%) | 3 (7.9%) |
| Child has emotional or behavioural needs† | 55 (53.9%) | 29 (82.9%) | 26 (66.7%) |
| Child needs special education† | 39 (38.2%) | 25 (71.4%) | 14 (35.9%) |

†Missing =2 participants; ‡Missing =3 participants; ADHD = Attention deficit/hyperactivity disorder; ASD = autism spectrum disorder; DCD = Developmental coordination disorder; CD = Conduct disorder; ODD = Oppositional defiant disorder.
